# A Systematic Review of Thiamine Supplementation in Improving Diabetes and Its Related Cardiovascular Dysfunction

**DOI:** 10.3390/ijms26093932

**Published:** 2025-04-22

**Authors:** Maria Serra, Rocco Mollace, Giovanna Ritorto, Sara Ussia, Carmen Altomare, Annamaria Tavernese, Mariaimmacolata Preianò, Ernesto Palma, Carolina Muscoli, Vincenzo Mollace, Roberta Macrì

**Affiliations:** 1 Pharmacology Laboratory, Institute of Research for Food Safety and Health IRC-FSH, Department of Health Sciences, University Magna Graecia of Catanzaro, 88100 Catanzaro, Italy; maria.serra@studenti.unicz.it (M.S.); giovanna.ritorto@studenti.unicz.it (G.R.); saraussia1598@gmail.com (S.U.); carmen.altomare@studenti.unicz.it (C.A.); muscoli@unicz.it (C.M.); mollace@libero.it (V.M.); 2 Department of Experimental Medicine, University “Tor Vergata” of Rome, 00133 Rome, Italy; 3 Department of Medicine and Surgery, University Campus Bio-Medico of Rome, 00128 Rome, Italy; an.tavernese@gmail.com; 4 Laboratory of Mass Spectrometry and Proteomics, Department of Health Sciences, “Magna Græcia” University, 88100 Catanzaro, Italy; preiano@unicz.it; 5 Veterinary Pharmacology Laboratory, Institute of Research for Food Safety and Health IRC-FSH, Department of Health Sciences, University Magna Graecia of Catanzaro, 88100 Catanzaro, Italy; palma@unicz.it; 6 Renato Dulbecco Institute, 88046 Lamezia Terme, Italy

**Keywords:** thiamine supplementation, hyperglycemia, cardiovascular disease (CVD), metabolic dysfunctions, endothelial dysfunction, hyperlactatemia

## Abstract

The significance of thiamine in human health is linked to its role in several pathways that control different disease processes. Significant improvements in cardiometabolic diseases, substantially impacted by thiamine imbalances, are observed with thiamine supplementation. Diabetic patients could see a reduction in cardiovascular (CV) risk due to thiamine’s significant impact on glucose metabolism. Specifically, increased ventricular filling pressures and oxygen consumption, indicative of CV dysfunction, are caused by oxidative and inflammatory damage to blood vessels, diabetic nephropathy, and elevated lactic acid production. Despite promising pre-clinical results for thiamine, clinical trials have yielded conflicting and contradictory findings due to limitations like small sample sizes and insufficient follow-up. To provide a summary of clinical study results, this systematic review assessed the impact of thiamine supplementation on diabetes and its CV complications. The studies included in this systematic review were retrieved from PubMed and Medline databases, in accordance with the Preferred Reporting Items for Systematic Reviews and Meta-Analyses (PRISMA) statement and following the Population Intervention Comparison Outcome (PICO) framework. Seven clinical studies were identified, which enlighten the association between thiamine supplementation, hyperglycemia, and cardiovascular disease (CVD). Although large-scale, multicenter studies with longer follow-up periods are needed, the association between thiamine and chronic metabolic dysfunction related to CV risk suggests its crucial role in preventing severe heart failure (HF).

## 1. Introduction

**Figure 1:**
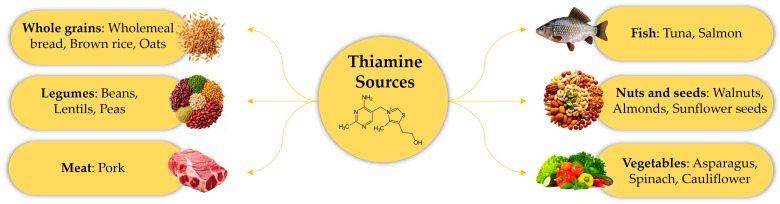
Dietary intake is the main source of vitamin B1 as it is present in many foods: Whole grains, pulses, meat, fish, nuts, seeds, and vegetables.

Thiamine (vitamin B1), a crucial micronutrient with a half-life of 1 to 3 weeks, is not synthesized by the body and is stored in limited concentrations [1]. Dietary intake, including whole grains (e.g., bread, brown rice, and oats), pulses (e.g., beans, peas, and lentils), meat (mainly pork), fish (e.g., tuna or salmon), nuts or seeds (e.g., walnuts, almonds, or sunflower seeds), and vegetables (e.g., asparagus, spinach, and cauliflower), and supplements are key sources of thiamine (Figure 1) [2].

**Figure 2:**
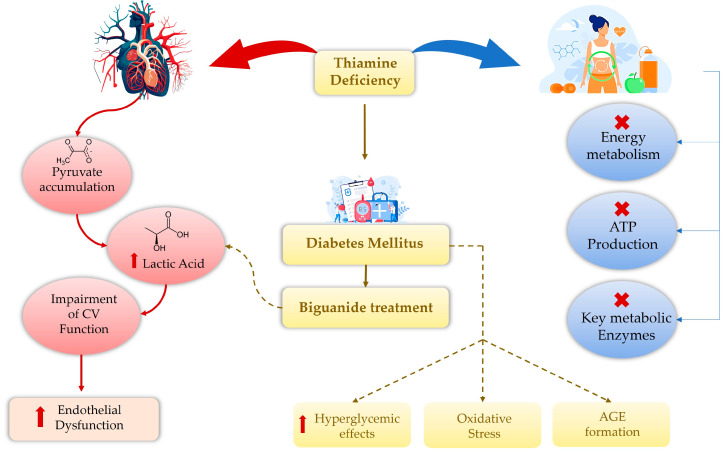
Thiamine deficiency triggers a cascade: pyruvate accumulates in the cardiovascular system, stimulating lactic acid production, compromising CV function, and leading to higher ventricular filling pressures, oxygen consumption, and endothelial damage. At the metabolic level, thiamine deficiency impairs energy metabolism, ATP generation, and key metabolic enzymes. Lack of thiamine intensifies diabetes, its hyperglycemia, oxidative stress, and the creation of AGEs. Cardiovascular (CV); advanced glycation end-product (AGE); adenosine triphosphate (ATP).

Thiamine occurs in three forms: thiamine monophosphate (TMP), thiamine pyrophosphate (TPP), and thiamine triphosphate (TTP) [3]; in the human body, TPP is the main form of thiamine and, after absorption, it is converted to its active form by thiamine pyro(di)phosphokinase [3]. TPP plays a role in multiple cellular enzyme functions involved in the metabolic processing of carbohydrates, lipids, and branched-chain amino acids (BCAAs). Pyruvate dehydrogenase (PDH), alpha-ketoglutarate dehydrogenase (α-KGDH), transketolase (TKT), and branched-chain α-ketoacid dehydrogenase (BCKD) all use TPP as a cofactor in glycolysis and oxidative carbohydrate decarboxylation [4]. Thiamine deficiency causes metabolic dysfunction, reduced energy, and damage to vulnerable organs, including the brain [5]. This results in impaired brain function, including glucose metabolism disruption, neurotransmitter imbalances, oxidative stress, acidosis, excitotoxicity, inflammation, endoplasmic reticulum stress, apoptosis, and a compromised blood–brain barrier [5]. In the cardiovascular (CV) system, thiamine deficiency results in elevated pyruvate levels, which increases lactic acid production, impairs function, and causes higher ventricular filling pressures and oxygen demand (Figure 2) [6].

Beyond its vital function in energy production, thiamine further catalyzes reactions within the pentose phosphate and hexose monophosphate pathways. The immune system’s function is also known to be impacted by thiamine.

Particularly, the closely related heme-independent oxygenase influences ICAM protein release, thereby modulating T cell activity [7]. Thiamine demonstrates anti-inflammatory properties (1) through antioxidant effects on neutrophils, macrophage protection against oxidative stress (by inhibiting the transcription factor NF-κB activation), and modulation of p53 activity (via p43 inhibition) [8].

### 1.1. Thiamine Deficiency

Thiamine deficiency frequently arises from a combination of predisposing factors in patients and low levels of thiamine may be caused by inadequate diet, impaired gut absorption, or higher metabolic demands; increased renal excretion is another possible cause of thiamine deficiency [9]. In the past, patients suffering from alcohol addiction, acquired immune deficiency syndrome (AIDS), and cancer were the most at-risk population for thiamine deficiency [9]. The risk of thiamine deficiency is significantly heightened by the increased metabolic requirements of pregnancy, lactation, hyperthyroidism, infection, critical illness, diabetes, or dialysis-dependent renal failure (RF) [10]. Furthermore, because of thiamine’s low bioavailability, post-bariatric surgery patients frequently suffer from thiamine deficiency [11]. Although a large part of the population has thiamine deficiency, its effects are more severe in high-risk groups. While some studies indicate that 25–31% of chronic alcoholics have thiamine deficiency, other evidence suggest that the rate is much higher (up to 80%) [12]. Thiamine deficiency in heart failure (HF) patients is prevalent in 21–98% of cases: advanced age, diuretic use, multiple comorbidities, and pregnancy all increase the likelihood of thiamine imbalance. Subclinical thiamine deficiency, detectable biochemically, could act as a CV and psychological stressor in active young adults.

### 1.2. Thiamine, Metabolic Dysfunctions, and CV Disease

A major risk factor for coronary heart disease (CHD) is diabetes mellitus (DM); a large population study conducted by Haffner et al. showed similar seven-year myocardial infarction (MI) incidence (fatal and non-fatal) in diabetics and non-diabetics with a history of MI [13,14].

A key role in carbohydrate metabolism is played by thiamine; in fact, diabetic thiamine deficiency leads to hyperglycemia-related cell damage, endothelial dysfunction, and oxidative stress. Lack of thiamine leads to altered glucose metabolism, a process that can result in damage to blood vessels. According to in vivo studies, reduced hexosamine pathway activity, and thus improved insulin sensitivity, may result from high-dose thiamine [15]. By reducing advanced glycation end-product (AGE) formation and mitigating hyperglycemia’s impact on cell replication, thiamine supplementation limits vascular damage from protein modification [15]. Endothelial dysfunction and heightened inflammation can be caused by the activation of protein kinase C (PKC). Reducing the activation of the polyol pathway in glucose metabolism through thiamine treatment has been shown to lower oxidative stress by reducing nicotinamide adenine dinucleotide phosphate hydrogen (NADPH)-mediated glutathione regeneration [16]. Moreover, a Cochrane review of randomized trials found a potential link between thiamine and lower albuminuria levels in patients with diabetic kidney disease (DKD). To summarize, thiamine might decrease glucose metabolism using other pathways, which could lead to higher oxidative stress and damage to the endothelium in people with diabetes [17].

Furthermore, a significant amount of research indicates that thiamine and its derivatives improve endothelial function and act as antioxidants in both cell cultures and animal models; these benefits appear in individuals with normal and high blood sugar [18]. An in vivo and clinical placebo-controlled pilot study showed that thiamine supplementation decreased urinary microalbumin. The results indicate a strong cardioprotective effect of thiamine, given the link between urinary microalbuminuria, which represent an early marker of diabetic nephropathy, and heightened cardiovascular disease (CVD) risk in both diabetic and non-diabetic patients [19]. Additionally, individuals homozygous for the risk allele showed lower erythrocyte thiamine levels and predicted CV features linked to thiamine deficiency (increased stroke volume, reduced vascular resistance, and heightened pressor responses), potentially associated with hypertension development and progression [20].

### 1.3. Thiamine Deficiency and HF

**Figure 3:**
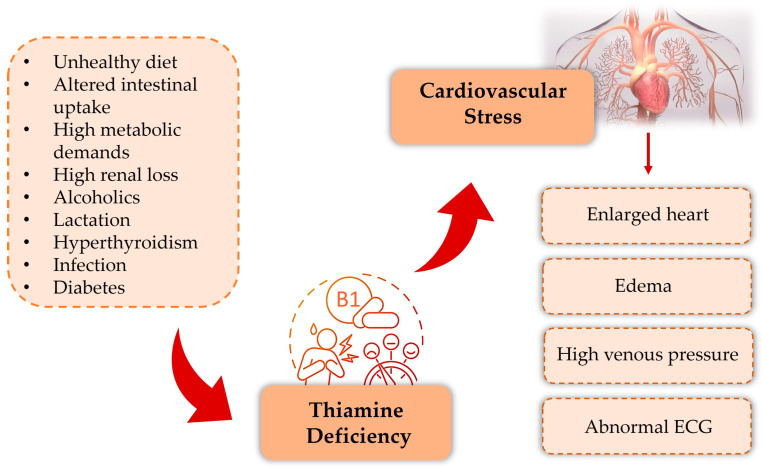
Several conditions can cause thiamine deficiency: insufficient nutrition, reduced intestinal uptake, elevated metabolic rate, high kidney loss, alcohol dependence, lactation, hyperthyroidism, infections, and diabetes. Enlarged heart, edema, high venous pressure, and abnormal ECGs mark thiamine-deficient HF.

A lack of thiamine is related to onset of wet beriberi, a form of HF, defined by elevated cardiac output alongside primarily right-sided heart failure and lactic acidosis [21]. Thiamine-deficient HF is marked by an enlarged heart (normal rhythm), edema, high venous pressure, neuropathy/pellagra, abnormal electrocardiogram (ECG), prolonged dietary deficiency, and thiamine-responsive symptoms/heart size reduction. The difficulty in diagnosis is often due to missing typical presentation features. Excessive cardiac muscle vasodilation and decreased peripheral vascular resistance, caused by impaired myocardial energy metabolism, lead to high HF output in beriberi [22,23]. Malnutrition, hyperthyroidism, age, hospitalization frequency, comorbidities, HF severity, and diuretic use commonly increase thiamine deficiency risk in HF patients (Figure 3) [24].

A consistent finding across multiple studies is that diuretics cause higher urinary thiamine excretion among HF patients, which may lead to thiamine deficiency [25]. However, the link between thiamine deficiency and diuretic use is still debated. In a study of 149 HF patients, a link between diuretic use and thiamine deficiency has been found. Higher doses of furosemide in healthy volunteers undergoing an uncontrolled intervention study led to greater thiamine loss in urine [26]. Intravenous furosemide frequently causes significant thiamine depletion in hypervolemic patients during hospitalization. HF patients treated with furosemide showed significantly reduced thiamine levels, unlike RF patients who did not experience a similar reduction; RF patients may experience less diuretic-induced thiamine loss, possibly because of lower excretion rates [27].

Although HF patients show variable thiamine levels, monitoring thiamine in those on chronic diuretics and supplementing, when necessary, remain worthwhile. Several groups have investigated how thiamine supplementation affects cardiac function in people with HF. In patients suffering from HF, thiamine might have a mild peripheral vasodilating effect, thus lowering afterload and boosting cardiac output [28,29]. Despite thiamine not being standard treatment for systolic HF, studies indicate it could improve left ventricular ejection fraction (LVEF) in these patients. In the end, a meta-analysis from Jain et al. demonstrated that HF patients exhibit a higher incidence of thiamine deficiency than controls [30]. Furthermore, preliminary findings from small observational studies and randomized controlled trials suggest a potential benefit of thiamine supplementation in improving ejection fraction (EF) and reducing symptoms in patients with HF [31]. Nevertheless, though thiamine supplementation is promising in improving cardiac functions, thiamine status, and HF-related symptoms, other evidence does not support the effects of thiamine supplementation in patients with HF [32].

The presence of some incomplete or conflicting data due to the bias introduced by small samples and the absence of suitable follow-up suggests that further investigations are still needed to better understand the role of thiamine supplementation in CVD.

## 2. Materials and Methods

### 2.1. Database Sources

This systematic review examines the relationship between thiamine and diabetes, using data from case reports, clinical trials (all phases), multicenter studies, and observational studies, to assess the impact of thiamine supplementation in diabetic adults with CV dysfunction. The key words used to search for articles were “thiamine and diabetes”, “thiamine supplementation and CV dysfunction”. The studies included in this systematic review were retrieved from PubMed and Medline databases, in accordance with the Preferred Reporting Items for Systematic Reviews and Meta-Analyses (PRISMA) statement and following the Population Intervention Comparison Outcome (PICO) framework. All papers written in English and published in the period from 1980 to 2025 were evaluated. Only articles published within the last 10 years were included in the qualitative analysis of this systematic review. The review protocol was registered with Prospero (PROSPERO 2025 CRD420251019098), available from https://www.crd.york.ac.uk/PROSPERO/view/CRD420251019098 (accessed on 25 March 2025).

### 2.2. Eligibility Criteria

Criteria for inclusion and exclusion are specified as follows: (a) human studies, (b) case reports; clinical trial; clinical trial protocol; clinical trial, phase I; clinical trial, phase II; clinical trial, phase III; clinical trial, phase IV; multicenter study; observational study, (c) adult+ 19 years represented the inclusion criteria; moreover, (a) animals, (b) review, systematic review, meta-analysis, (c) child: birth–18 years have been selected for exclusion criteria.

### 2.3. Study Outcomes

The significant impact of thiamine on glucose metabolism could reduce CV risk in diabetic patients. The disruption of normal CV function, manifested by increased ventricular filling pressures and oxygen consumption, is a known consequence of blood vessel oxidative and inflammatory injury, diabetic nephropathy, and elevated lactic acid production. 

The aim of this systematic review was to evaluate the impact of thiamine supplementation on diabetes and the related CV dysfunction through clinical studies to provide an overview of the results. To achieve this objective, we examined several clinical studies that collected the effects of thiamine supplementation in patients with diabetes. The correlations between the reduction of hyperglycemia and the adverse effects on endothelial dysfunction and CV dysfunction have been analyzed.

### 2.4. Statistical Analysis

A qualitative summary of results across comparable exposure groups was made from different papers.

## 3. Results

### 3.1. Data Collection

**Figure 4:**
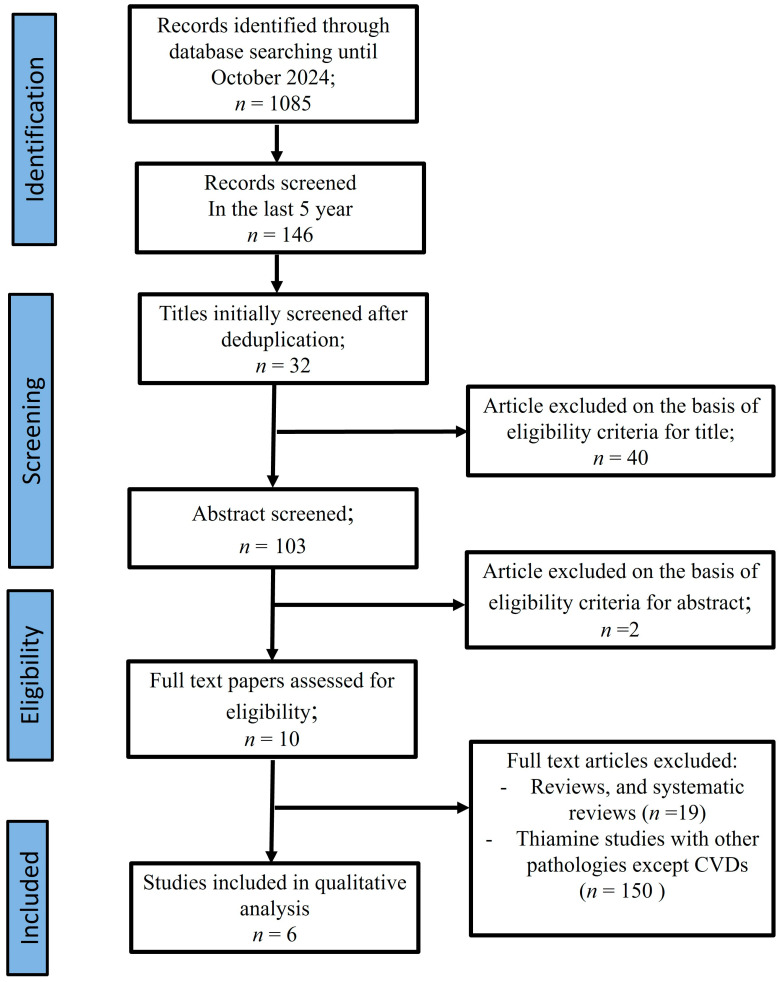
PRISMA Flow Chart.

A literature search from 1980–2025 (conducted in February) identified 100 records. Following abstract and full-text screening, we limited the search to the past 10 years, identifying 60 PubMed and 50 Medline articles. Subsequently, we removed the duplications, and 60 papers were screened for abstract; 26 were excluded due to keywords present in their abstracts and 34 full-text papers were assessed for eligibility. On the basis of exclusion criteria, we excluded 5 articles because they concern animals, 5 as they were reviews, systematic reviews, and meta-analyses, and 12 because they involved patients under 18 years of age. Finally, one paper has been excluded for language and eight papers were included in the qualitative analysis (Figure 4).

### 3.2. Thiamine Supplementation and Diabetes

The main features of the eight articles on supplementation of thiamine in patients with diabetes are summarized in Table 1.

The reviewed studies reported that thiamine supplementation is clearly associated with better diabetes symptoms and features. The clinical studies showed a strong correlation between lower blood sugar and a significant reversal of diabetes-related CV dysfunctions.

The effectiveness of a personalized oral supplement (ALA, carnosine, and thiamine) in combating CVD in T2DM patients, related to increased oxidative stress, was investigated via a randomized, double-blind, placebo-controlled trial. A daily supplement of 7 mg ALA/kg, 6 mg carnosine/kg, and 1 mg thiamine/kg body weight, or a placebo, was given to 82 randomly assigned obese T2DM patients for 8 weeks. High risks of microvascular and macrovascular complications existed for these patients due to their obesity, T2DM (indicated by abnormal HbA1c), and dyslipidemia (fulfilling metabolic syndrome criteria). Personalized supplementation with ALA, carnosine, and thiamine significantly reduced glucose in individuals with type 2 diabetes, potentially through increased insulin production. Minimal changes in insulin sensitivity resulted from supplementation, as increased insulin levels were counteracted by a small decrease in glucose [33].

The second study’s findings suggest that hyperglycemia’s negative consequences are potentially increased by hypertension and dyslipidemia. This research examined the impact of high-dose thiamine on blood pressure, serum lipids, and hs-CRP in individuals with impaired glucose metabolism. A double-blind, randomized, crossover trial was conducted on 12 hyperglycemic participants (10 with impaired glucose tolerance and 2 with newly diagnosed T2DM), who received both placebo and 300 mg of thiamine daily for six weeks. Participants receiving six weeks of thiamine supplements had significantly reduced DBP levels compared to baseline and week 3 measurements. In the placebo arm, these variables remained constant. Assessments of lipid profiles and hs-CRP revealed no significant differences between the supplement and placebo groups. High-dose thiamine may benefit early hyperglycemia patients by improving blood pressure and reducing vascular alterations [34].

In a large urban community hospital, a retrospective observational study was conducted on interventional bariatric surgery patients, with a body mass index of at least 35 kg/m^2^, recruited from 2013 to 2015. This study reported thiamine deficiency among patients who underwent gastric bypass surgery. Complex obesity may often involve thiamine deficiency, possibly linked to diabetes, a suggestion has been made. Traditionally, clinicians have largely associated clinical thiamine deficiency with alcoholic patients exhibiting cardiovascular or neuropsychiatric symptoms. Clinical thiamine deficiency is diagnosed by the presence of consistent clinical symptoms and either low whole-blood thiamine or improvement/resolution of symptoms following thiamine supplementation.

With 101 individuals excluded for prior bariatric surgery or heavy alcohol use, the final study population consisted of 400 patients. Of 400 patients, 66 had clinical thiamine deficiency; among those, 9 showed gastrointestinal symptoms, 46 cardiac symptoms, (such as extremity edema and dyspnea with minimal exertion), 39 neurological symptoms, and 3 neuropsychiatric symptoms. Clinical thiamine deficiency is common in this group, and a higher BMI is associated with increased risk. Thiamine supplementation led to the most improvement in lower extremity edema (28/43 participants) among those who reported significantly better, lasting clinical symptoms. Improvements in numbness/paresthesia (26/43), dysphagia (5/43), and dyspnea with minimal exertion (4/43) were common. Repleting an element after its low intake has caused a harmful pathophysiological condition shows nutritional benefit by improving health. Supplements can be pharmacologically beneficial by improving conditions unrelated to nutritional deficiencies. However, a prospective study is needed to validate these findings and investigate the mechanisms of thiamine deficiency in obese individuals with pathological conditions [35].

The role of thiamine supplementation in AHF patients was evaluated in a pilot clinical trial. On the first and second evenings, subjects received either 100 mg of thiamine or a placebo and outcome measures were obtained 8 h after drug infusion. Through a 100 mm visual analog scale (VAS), dyspnea was measured in three positions: sitting up with oxygen, sitting up without oxygen, and lying down without oxygen, with 0 representing no dyspnea. Reduced dyspnea in hospitalized patients, potentially from adding thiamine to their usual treatment, was the primary outcome measure.

Secondary outcome measures included peak expiratory flow rate, type B natriuretic peptide (BNP), free fatty acids, glucose, hospital length of stay, and 30-day rehospitalization. From 130 randomized participants, data from 118 (55 control, 63 treatment) were analyzed; 89% of both groups presented primarily with AHF. Treatment significantly increased thiamine levels, while control group levels stayed the same. One patient was deficient in thiamine. Significant differences between groups over time were only observed in upright, oxygen-treated patients’ dyspnea. It was suggested that thiamine, in addition to standard care, would improve dyspnea in hospitalized individuals with AHF.

Instead, standard thiamine doses failed to improve dyspnea, biomarkers, or other clinical parameters in patients with mild to moderate AHF without thiamine deficiency [36].

A case report describes a 75-year-old woman’s hospital referral for sudden-onset shock of unknown cause and severe lactic acidosis. A year before her presentation, the patient had a stroke affecting the left side of her body and causing mild cognitive problems. Buformin (100 mg daily) and sitagliptin (50 mg daily) were used to treat her DM. In addition, she received cilostazol (100 mg daily) to prevent further strokes, along with lansoprazole (30 mg daily) and magnesium oxide (660 mg daily).

The patient has been treated with biguanide for T2DM and two key clinical issues emerged from this case: high-dose intravenous thiamine is possibly vital for treating thiamine deficiency in diabetic patients on biguanide, regardless of other causes.

This is the first case study of biguanide-induced lactic acidosis complicated by thiamine deficiency in a diabetic patient lacking typical thiamine deficiency risk factors. Thiamine deficiency, biguanide toxicity, or a combination thereof may explain the patient’s lactic acidosis. In second place, high-dose intravenous thiamine infusion is helpful in treating this disorder and provides valuable diagnostic information. Characterized by ventricular systolic dysfunction and intense vasodilation causing circulatory collapse, cardiac beriberi is a severe manifestation of thiamine deficiency. Patients showed this cardiac dysfunction as low cardiac output. The “metabolic resuscitator” role of thiamine in critically ill patients is a subject of considerable attention. Supplementation with thiamine is a generally safe and well-tolerated treatment, with rare side effects like anaphylaxis. Therefore, biguanide-associated lactic acidosis patients should be evaluated for thiamine deficiency and treated promptly with high-dose thiamine as a diagnostic measure [37].

A different case report highlighted a 25-year-old woman’s isolated megaloblastic anemia (normal blood counts alongside insulin-dependent diabetes, severe bilateral sensorineural hearing loss, and pigmentary retinopathy), all of which suggested a diagnosis of TRMA. Thiamine treatment (100 mg/day) achieved normoglycemia and normalized hemoglobin. Although stopping insulin was an option, childhood/adolescent psychotic, depressive, and personality disorders necessitated antipsychotics. High fasting blood sugar led to starting lifelong insulin therapy. The patient needed daily insulin injections and started taking 100 mg of thiamine orally every day in childhood. On admission, a single platelet concentrate transfusion was given. Treatment started with 300 mg of parenteral thiamine three times daily for the first 3 days, followed by 300 mg once daily orally. Thiamine supplementation resulted in increases in reticulocytes (80%, 223 × 10^9^/L), hemoglobin (9.6 g/dL), leucocytes (7.6 × 10^9^/L), and platelets (219 × 10^9^/L) within one week. Partial correction of the anemia likely eliminated dyspnea, which was probably caused by its rapid onset. There was no change in insulin requirements. On day 7, the patient was discharged; however, their oral thiamine supplementation (300 mg daily) persisted. Normal blood cell counts were observed three weeks post-treatment initiation. This thiamine supplementation plan (300 mg parenterally three times daily for 3 days, then 300 mg orally daily) follows the Wernicke encephalopathy protocol. It is hypothesized that administering high-dose thiamine parenterally and rapidly could promptly stimulate blood cell regeneration. However, thiamine failed to improve the patient’s deteriorating pancreatic endocrine insufficiency. This aligns with earlier studies showing that patients with TRMA typically require insulin treatment [38].

The latest case report describes a 19-year-old man with TRMA who arrived at the emergency room with increasing shortness of breath, chest pain, and paleness. Diagnosed megaloblastic anemia, insulin-dependent DM, and severe bilateral sensorineural hearing loss in the patient suggest possible TRMA. A daily 300 mg oral dose of thiamine resulted in normalized hemoglobin. Elevated glucose necessitated a three-times-daily fixed insulin treatment regimen. The classic triad of megaloblastic anemia, DM, and sensorineural deafness, diagnosed in childhood, was reflected in the patient’s medical history and initial presentation, consistent with other reported cases. Intravenous thiamine (100 mg daily for 10 days) began on day 1; then, oral thiamine (300 mg daily) started. Thiamine supplementation resulted in increased reticulocytes, hemoglobin, and platelets within 10 days. The dyspnea and chest pain, likely due to rapidly developing anemia, resolved after some improvement in the anemia, but insulin needs remained constant. Oral thiamine supplementation persisted after the patient’s discharge on day 7. Following three weeks of treatment, blood cell counts reached normal levels. Therefore, pharmacological thiamine treatment effectively treats megaloblastic anemia and DM [39].

## 4. Discussion

This systematic review provides a report on the effect of supplementation with thiamine in patients with diabetes. The collection of results from the last 10 years (2015–2025) offer a broader perspective on the results obtained, which is useful in the clinical setting for the creation of nutraceutical targeted therapies improving the patient’s quality of life. The impact of thiamine supplementation on diabetes symptoms and features, including the correlation with CVD, has been evaluated. Indeed, preliminary data from pre-clinical and clinical studies show thiamine supplementation significantly affects CVD. A fast onset of thiamine deficiency can result from various factors such as poor diet, digestive issues, alcohol misuse, cancer, diabetes, pregnancy, breastfeeding, and hyperthyroidism [18,40]. Human thiamine levels are altered by genetic mutations impacting thiamine transporters: alterations affecting solute carrier family 19-member 2 (SLC19A2) may lead to TRMA, sensorineural hearing loss, hyperglycemia, and diabetes mellitus (DM) [41]. Furthermore, diabetics’ hyperglycemia and metabolic problems may be exacerbated by common thiamine deficiency. A lack of thiamine disrupts energy metabolism, adenosine triphosphate (ATP) production, and key metabolic enzymes, resulting in lower ATP levels, oxidative stress, and cell death. Additionally, untreated thiamine deficiency leads to serious malnutrition and weight loss due to prolonged energy shortfalls [5]. Insufficient TPP causes PDH to be inactive, blocking the conversion of pyruvate to acetyl-coenzyme A (acetyl-CoA) and the use of carbohydrates in the Krebs cycle. Lactic acidosis and reduced mitochondrial ATP production occur due to anaerobic pyruvate accumulation and its conversion to lactic acid [42].

Concurrently, chronic vascular inflammation leads to endothelial dysfunction, a major factor in atherosclerosis and CVD progression, substantially increasing the risk of heart disease [43].

High glucose negatively affects endothelial cells; however, this effect is mitigated by thiamine’s reduction of protein glycation [44]. Specifically, adequate thiamine is critical for proper carbohydrate metabolism; its deficiency worsens diabetes and its related complications (e.g., endothelial dysfunction, oxidative stress, AGE formation, cellular damage), ultimately causing cellular injury [45,46].

Maintaining adequate thiamine levels might lower the risk of hypertension, heart attacks, angina, type 2 diabetes mellitus, hypercholesterolemia, and depression. Particularly, lower HbA1c and fasting glucose were observed with adequate thiamine consumption [47].

A cost-effective way to improve heart function, particularly in in elderly HF patients with low LVEF, may be thiamine supplementation [48]. Growing interest in thiamine is due to findings suggesting it may lessen diabetes-related issues. Indeed, recent evidence indicates a potential for thiamine to mitigate the detrimental effects of hyperglycemia on endothelial cells.

The accelerated atherosclerosis (impaired endothelium-dependent vasodilation and CVD) seen in DM suggests that better glycemic control could greatly impact microvascular and macrovascular dysfunction, as indicated by studies [49,50]. Thiamine supplementation might promote better endothelial function and slower progression of atherosclerosis. This finding may significantly impact healthcare, especially in the CV field. Furthermore, because thiamine acts as a cofactor in their fast metabolism, supplementing it could impact cancer cell metabolism [51]. This might also decrease doxorubicin-induced cardiotoxicity (including lipid peroxidation, DNA damage, mitochondrial dysfunction, and ROS generation), resulting in better echocardiogram outcomes [52].

Based on the evidence above, we aimed to analyze recent clinical trials to verify a potential link between thiamine supplementation and hyperglycemia; while limited, these trials may offer key insights.

The clinical studies analyzed showed a strong correlation between lower blood sugar related to thiamine supplementation and a significant reversal of diabetes-related CV dysfunctions.

Oral thiamine supplementation could improve symptoms and quality of life in patients with diabetes without appreciable adverse effects, thus apporting a significant beneficial effect on related endothelial dysfunction and CV and neurological complications.

Despite promising pre-clinical results showing thiamine’s impact on oxidative stress, inflammation, and cardiovascular dysfunction (excluding heart rate), its effect on HF patients remains inconclusive. The limitations of existing thiamine studies include small sample sizes, indirect thiamine concentration measures, methodological inconsistencies, impractical supplementation approaches, a patient population limited to hospitalized individuals, and the absence of a reliable cardiac function assessment method.

This systematic review emphasizes thiamine’s crucial role in high blood sugar control, especially for patients at risk of micro- and macrovascular complications [33]. Furthermore, high-dose B1 supplementation reduced diastolic blood pressure in hyperglycemic patients, thus confirming its protective role in endothelial function [34]. In obese patients undergoing bariatric surgery, thiamine supplementation plays a crucial role in reducing vascular complications (edema) and improving cardiorespiratory function by reducing dyspnea [35]. The importance of thiamine in reducing shortness of breath for AHF patients with thiamine deficiency supports the idea that thiamine replacement could improve CV function [36,53]. The “metabolic resuscitator” potential of thiamine is suggested by case reports showing it may counteract biguanide-induced lactic acidosis in individuals with T2DM and thiamine deficiency [37]. Indeed, mortality risk is increased in AHF patients exhibiting hyperlactatemia and acid–base disturbances [54], with elevated lactate and reduced pH being additional mortality predictors in cardiogenic shock [55]. Therefore, thiamine might be essential in correcting acid–base imbalance in cases of chronic metabolic decompensation.

## 5. Conclusions

The association of thiamine with chronic metabolic dysfunction linked to CV risk suggests its crucial role in preventing severe HF outcomes.

Despite the encouraging evidence, the reviewed studies share limitations such as small sample sizes, indirect thiamine measurement, inconsistent methods, impractical supplementation, a focus on hospitalized patients, and a lack of robust cardiac assessments.

These findings should be interpreted cautiously due to low data counts. Further clinical trials are necessary to validate these findings and explore how nutritional requirements shift with chronic illnesses and the clinical advantages of supplementation. These results need validation through large, randomized, double-blind, placebo-controlled, multicenter studies examining thiamine’s effect on metabolic problems linked to major CV events in patients with HF.

## Figures and Tables

**Table 1:** The table shows the selected articles, specifying the clinical trial aims, databases used, and results.

Authors, Year	Aim of Studies	Search Databases	Types of Studies Included	Summary of Results	Ref.
Karkabounas, S. et al., 2018	Examination of the efficiency of an individualized oral supplementation with carnosine, α-lipoic acid (ALA) corrosive, and thiamine in patients with type 2 diabetes mellitus (T2DM) associated with different complications, particularly CVD.	PubMed, Medline.	Randomized, double-blind, placebo-controlled trial.	Decreasing glucose concentration in T2DM patients throughout potentially enhanced insulin release from the pancreas. The supplement had only a minor impact on insulin sensitivity because the considerable increase in insulin was balanced by a minor but prominent reduction in glucose.	[33]
Alaei-Shahmiri, F. et al., 2015	Estimation of the effects of high-dose thiamine on blood pressure, high-sensitivity C-reactive protein (hs-CRP), and serum lipids in individuals with damage glucose metabolism.	PubMed, Medline.	Double-blind, randomized, crossover trial.	Significant decreasing of diastolic blood pressure (DBP) in participants consuming thiamine supplements for six weeks relative to baseline and a predisposition upon a lower systolic blood pressure (SBP) at week six corresponding to baseline. This deviation was not modified in the placebo groups. No relevant variations were highlighted in the supplement or placebo patient treatment when lipid profile and hs-CRP were measured. High-dose thiamine supplementation may have advantageous effects on the blood pressure of individuals with hyperglycemia.	[34]
Nath, A. et al., 2017	Evaluation of patients with a body mass index of at least 35 kg/m^2^ in pre-operative gastrointestinal bariatric clinic from 2013 to 2015. Patients have been estimated for clinical symptoms.	PubMed, Medline.	Retrospective, observational, single-institution study.	An intensified improvement was observed in the edema of the lower extremities (28/43 participants) among reporting significantly improved and lasting clinical symptoms subsequent to thiamine supplementation. Significant advancements in dysphagia, numbness/paresthesia, and dyspnea with minimal exertion were prevalent.	[35]
Smithline, H.A. et al., 2018	Evaluation of the role of thiamine supplementation in acute heart failure (AHF) patients.	PubMed, Medline.	Stratified block randomized, double-blind, placebo, pilot, controlled study.	A significant difference was observed only for the measurements of oxygen and dyspnea while sitting between the two groups in the study. Standard thiamine dose supplementation was not demonstrated to improve dyspnea, biomarkers, or other clinical parameters in patients with mild to moderate AHF without thiamine deficiency.	[36]
Godo, S. et al., 2017	A case report was conducted to evidence two crucial scientific aspects. Administration of high-dose intravenous thiamine can be uncertain in diabetic sufferers on biguanide in thiamine deficiency, regardless of various contributing factors. Secondly, high-dose intravenous thiamine infusion is advantageous to treating diabetic patients and suggests essential diagnostic information.	PubMed, Medline.	Case reports.	Thiamine supplementation was considered a generally safe and well-tolerated treatment with the progression of rare side effects like anaphylaxis. Accordingly, patients suffering from lactic acidosis associated with biguanide should be observed and evaluated for thiamine deficiency and treated immediately with high doses of thiamine as a diagnostic measurement.	[37]
Moulin, V. et al., 2018	Analysis of a 25-year-old woman with isolated megaloblastic anemia (thrombocyte and leucocyte counts were normal), severe bilateral sensorineural hearing loss, insulin-dependent DM, and bilateral pigmentation retinopathy, which led to a suspicion of thiamine-responsive megaloblastic anaemia (TRMA).	PubMed, Medline.	Case reports.	Thiamine treatment (100 mg daily) resulted in normoglycemia and normalization of hemoglobin levels, in particular permitting temporary discontinuation of insulin treatment. Parenteral and successive oral thiamine supplementation increased reticulocytes to 80% (223 × 10^9^/L), haemoglobin level to 9.6 g/dL, leucocytes to 7.6 × 10^9^/L, and platelets to 219 × 10^9^/L. Probably caused by the rapid onset of anemia, dyspnea disappeared once anemia was fractionally rectified. The insulin requirements did not change, although in the patient a progression of pancreatic endocrine insufficiency over time was observed and there is no pancreatic endocrine response to thiamine supplementation.	[38]
Nouira, N. et al., 2020	A report case analyzed a 19-year-old man suffering from TRMA with insulin-dependent DM and severe bilateral sensorineural hearing loss, presenting in the emergency department with bicytopenia (haemoglobin 5.4 g/dL, thrombocytes 38 × 10^9^/L) revealed by dyspnea and chest pain.	PubMed, Medline.	Case reports.	Significant increases in reticulocytes, hemoglobin level, and platelets were observed within 10 days of thiamine supplementation. Extinction of dyspnea and chest pain, probably due to rapid anemia, once the anemia disappeared. Blood cell count restored after 3 weeks of thiamine supplementation. Improvement of megaloblastic anemia and DM by pharmacological treatment with thiamine.	[39]

AHF: Acute heart failure; ALA: α-lipoic acid; CVD: cardiovascular disease; DBP: diastolic blood pressure; DM: diabetes mellitus; hs-CRP: high-sensitivity C-reactive protein; SBP: systolic blood pressure; T2DM: type 2 diabetes mellitus; TRMA: thiamine-responsive megaloblastic anemia.

## Data Availability

No new data were created or analyzed in this study.

## Abbreviations

Acetyl-CoA	acetyl coenzyme A
AGEs	advanced glycation end-products
AHF	acute heart failure
AIDS	acquired immune deficiency syndrome
ALA	α-lipoic acid
α-KGDH	alpha-ketoglutarate dehydrogenase
ATP	adenosine triphosphate
BCAAs	branched-chain amino acids
BCKD	branched-chain α-ketoacid dehydrogenase
BNP	type B natriuretic peptide
CHD	coronary heart disease
CV	cardiovascular
CVD	cardiovascular disease
DBP	diastolic blood pressure
DKD	diabetic kidney disease
DM	diabetes mellitus
DNA	deoxyribonucleic acid
ECG	electrocardiogram
EF	ejection fraction
HbA1c	hemoglobin A1c
HF	heart failure
hs-CRP	high-sensitivity C-reactive protein
ICAM	intercellular adhesion molecule
LVEF	left ventricular ejection fraction
MI	myocardial infarction
NADPH	nicotinamide adenine dinucleotide phosphate hydrogen
PDH	pyruvate dehydrogenase
PICO	Population Intervention Comparison Outcome
PKC	protein kinase C
PRISMA	Preferred Reporting Items for Systematic Reviews and Meta-Analyses
RF	renal failure
ROS	oxygen reactive species
SBP	systolic blood pressure
SLC19A2	solute carrier family 19 member 2
T2DM	type 2 diabetes mellitus
TKT	transketolase
TMP	thiamine monophosphate
TPP	thiamine pyrophosphate
TRMA	thiamine-responsive megaloblastic anemia
TTP	thiamine triphosphate
VAS	visual analog scale
